# Temporal Characteristics of Ozone (O_3_) in the Representative City of the Yangtze River Delta: Explanatory Factors and Sensitivity Analysis

**DOI:** 10.3390/ijerph20010168

**Published:** 2022-12-22

**Authors:** Yu Lu, Zhentao Wu, Xiaobing Pang, Hai Wu, Bo Xing, Jingjing Li, Qiaoming Xiang, Jianmeng Chen, Dongfeng Shi

**Affiliations:** 1 College of Environment, Zhejiang University of Technology, Hangzhou 310023, China; 2 National Institute of Metrology, Beijing 102200, China; 3 Shaoxing Ecological and Environmental Monitoring Center of Zhejiang Province, Shaoxing 312000, China; 4 Hangzhou Xufu Detection Technology Co., Ltd., Hangzhou 310023, China

**Keywords:** ozone, peroxyacetyl nitrate, temporal variation, GAM, OBM, Yangtze River Delta

## Abstract

Ozone (O_3_) has attracted considerable attention due to its harmful effects on the ecosystem and human health. The Yangtze River Delta (YRD), China in particular has experienced severe O_3_ pollution in recent years. Here, we conducted a long-term observation of O_3_ in YRD to reveal its characteristics. The O_3_ concentration in autumn was the highest at 72.76 ppb due to photochemical contribution and local convection patterns, with its lowest value of 2.40 ppb in winter. O_3_ exhibited strong diurnal variations, showing the highest values in the early afternoon (15:00–16:00) and the minimum in 07:00–08:00, specifically, peroxyacetyl nitrate (PAN) showed similar variations to O_3_ but PAN peak usually occurred 1 h earlier than that of O_3_ due to PAN photolysis. A generalized additive model indicated that the key factors to O_3_ formation were NO_2_, PAN, and temperature. It was found that a certain temperature rise promoted O_3_ formation, whereas temperatures above 27 °C inhibited O_3_ formation. An observation-based model showed O_3_ formation was VOCs-limited in spring and winter, was NO_x_-limited in summer, and even controlled by both VOCs and NO_x_ in autumn. Thus, prevention and control strategies for O_3_ in the YRD are strongly recommended to be variable for each season based on various formation mechanisms.

## 1. Introduction

O_3_ is a typical secondary pollutant with a complex formation mechanism involving a series of chemical reactions among volatile organic compounds (VOCs), oxides of nitrogen (NO_x_, NO + NO_2_) and carbon monoxide (CO) [1]. O_3_, as an important indicator of photochemical pollution, plays a central role in the oxidation of chemical and climate-relevant trace gases in the troposphere [2]. O_3_ pollution has become a serious air quality problem affecting human health, vegetation, biodiversity, and climate worldwide as O_3_ concentrations have increased significantly since the second half of the 20th century [3,4]. According to a government report in China in 2020, O_3_ is the only air pollutant that maintained a rising trend during the last 5 years and O_3_ pollution is another urgent environmental problem in China, except for haze [5].

It has been noted that O_3_ levels increased by 30% to 70% in the temperate and polar regions of the Northern Hemisphere from 1896–1975 [3]. Despite policies to reduce precursor emissions, O_3_ concentrations have remained high; therefore, in-depth studies of the factors influencing O_3_ formation are critical to controlling ozone pollution. In addition to precursor substances, meteorological factors also have an influential effect on ambient O_3_ concentration [6,7]. Peroxyacetyl nitrate (CH_3_C(O)O_2_NO_2_, PAN) in the atmosphere serves also as a reliable and scientific indicator of photochemical pollution [8]. PAN acts as a temporary reservoir for NO_x_ and radicals, which can be transported to distant areas to redistribute NO_x_ as well as influence O_3_ production on a regional or even global scale [9]. Recently, some related studies focused on severe photochemical smog events in China with a relatively short period of measurement [10,11], but most of them focused on the events occurring in Beijing and the Pearl River Delta (PRD), China [10,12,13].

The Yangtze River Delta (YRD) is the region with the highest degree of urbanization and industrialization in China, and the coal-based energy system not only supports urbanization and industrialization but also contributes to serious regional air pollution problems [14]. Since 2017, O_3_ has become the most significant air pollutant in the YRD [5]. As the integrated development of the YRD has become a national strategy [15], the new situation of air pollution prevention and control makes it necessary to conduct an in-depth study on the O_3_ characteristics in the YRD to promote the sustainable development of the YRD.

So far, researchers have studied the spatial and temporal variations of O_3_, the mechanism of its formation, and the influencing factors [16,17,18]. Previous research has shown that O_3_ in the upper troposphere has increased annually across Europe from 1995 to 2013 [19]. In the troposphere with little UV radiation, it has been widely established that NO_2_ photolysis at wavelengths ≤424 nm becomes the main source of atomic oxygen and contributes to O_3_ formation. The main feature of O_3_ formation is the nonlinear dependence of O_3_ production on its precursors, i.e., NO_x_ and VOCs [2]. Several studies have proven that there is a complex photochemical interaction between O_3_ and PM _2.5_ and that PAN photochemistry has both negative and positive effects on O_3_ production [2,9]. In addition, some studies have found that the correlation between O_3_ and meteorological factors varies by season and region [16].

Generally, the available literature provides an essential foundation for ozone research. However, many studies focused on a single air pollutant, and few considered the synergistic and coordinated effects of multiple pollutants in a comprehensive manner [15]. Thus, in this study, we performed a one-year continuous observation of O_3_, PAN, other pollutants, and meteorological parameters to provide further insights into the formation mechanism of ambient O_3_ in the YRD, China in 2021 in a typical city located in the YRD, China, Shaoxing city, which is the core city of the Great Hangzhou Bay Area, near Shanghai and Hangzhou [20]. Here, a generalized additive model (GAM) was used to synthetically quantify the complex nonlinear relationships between O_3_ and multiple parameters, specifically including PAN for the first time. Compared with machine learning techniques, the GAM can uniquely quantify trends in O_3_ concentrations, which is better for understanding and controlling pollution [16]. Additionally, an observation-based model (OBM) was used to investigate the sensitivity of O_3_ production in different seasons, evaluating the effects of precursor reduction on O_3_ production. Therefore, this study can provide a comprehensive understanding of O_3_ formation and scientific evidence for the prevention and control of O_3_ pollution in the YRD, China.

## 2. Materials and Methods

### 2.1. Observation Site

**Figure 1:**
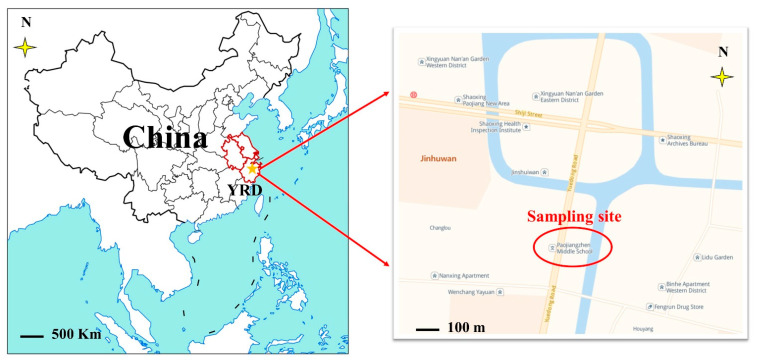
The sampling site in Yangtze River Delta (YRD) in China (**left**) and the location of the sampling site in Shaoxing city (**right**).

A field observing campaign was continuously conducted from January–December 2021 at an Atmospheric Observation Supersite (120.62° E, 30.08° N) in Shaoxing shown in Figure 1, located on the rooftop of an approximately 15 m-high building. The observation site is surrounded by residential areas and administrative offices, with well-developed traffic and no obvious industrial pollution sources, which can be considered as a typical urban site in the YRD, China. March, April, and May are considered as spring season, June, July, and August as summer, September, October, and November as autumn, and December, January, and February as winter in this paper according to the climate in YRD, China.

### 2.2. Measurement Apparatus and Methods

Concentrations of atmospheric O_3_, NO_x_, SO_2_, and CO were measured by instruments (USA i-series 49i, 42i, 43i, and 48i, Thermo Fisher Scientific, Waltham, MA, USA), while PM_2.5_ is sampled on a tapered element oscillating microbalance (TEOM1405, Thermo Fisher Scientific, Waltham, MA, USA). We calibrated each of these instruments periodically on a monthly basis. Meteorological data (temperature (T) and relative humidity (RH)) were obtained from an on-site meteorological station.

PAN concentration was determined through a PAN analyzer (PAN, Met Con Inc., SN, German) containing gas chromatography with an electron capture detector (GC-ECD), a sampling and calibration unit, and a computer control unit. It is the reaction of NO and acetone under UV light to produce PAN standard gas. During the observation period, calibration was performed once a week. The PAN was detected every 5 min with a detection limit of 50 ppt. The overall uncertainty of the measurement was estimated to be ± 3%.

Ambient VOCs were measured online by a cryogen-free automated gas chromatography (GC) system equipped with a flame ionization detector (FID) and mass spectrometer detector (MSD) with a temporal resolution of 1 h (Lu et al., 2022). A total of 94 VOC components were identified and measured during the course of this study. Detailed descriptions of the principles, performance, quality assurance, and quality control (QA/QC) processes of the online GC-MS/FID system are available in a previously published paper [20].

### 2.3. Generalized Additive Model

GAM, an extension of the additive model, is a flexible and free regression model that can make more reasonable nonlinear fittings than traditional statistical models [21]. It is widely used to reveal the complex nonlinear relationships between air pollutants and contributing factors in some air pollution studies [22,23]. In this study, GAM was applied to analyze the relationship between O_3_ and some factors including PAN, VOCs, PM_2.5_, NO, NO_2_, CO, T, and RH, respectively. Its basic form is as follows [24]:

(1) $$g\left(\mu \right)=\alpha +f_{1}\left(x_{1}\right)+f_{2}\left(x_{2}\right)+\cdots f_{n}\left(x_{n}\right)+\beta$$

where μ is the response variable; $g\left(\mu \right)$ is the “link” function; α is the intercept; $x_{1}$, $x_{2}$, and $x_{n}$ are the impact factors; $f_{1}\left(x_{1}\right)$, $f_{1}\left(x_{1}\right)$, and $f_{n}\left(x_{n}\right)$ are the smooth functions of the impact factors; and β is the residual.

### 2.4. Observation-Based Model

An observation-based model (OBM) was used to simulate the net O_3_ production rate and the sensitivity mechanism of O_3_ production in this study [25]. The model is informed by observations of 94 VOCs, trace gases (O_3_, NO_x_, and CO), and the meteorological parameter as boundary conditions for simulating atmospheric photochemical processes. The relative incremental response (RIR) was calculated using Equation (2) to evaluate the relative contribution of the precursors to O_3_ formation [18]:

(2) $$RIR\left(X\right)=\frac{\frac{\left[P_{O_{3}}\left(X\right)-P_{O_{3}}\left(X-\Delta X\right)\right]}{P_{O_{3}}\left(X\right)}}{\frac{\Delta S\left(X\right)}{S\left(X\right)}}$$

where, X represents a specific precursor of O_3_, including VOCs, NO_x_, and CO, respectively. $P_{O_{3}}$ is the O_3_ formation potential from 07:00 a.m. to 19:00 p.m., which is the net amount of O_3_ production rate during the evaluation period and can be obtained from the OBM; ΔX represents the change in X concentration; $S\left(X\right)$ means the observed concentrations of species X, which represents the combined impacts of regional traffic and on-site emissions; $\frac{\Delta S\left(X\right)}{S\left(X\right)}$ means the relative change of $S\left(X\right)$ [26].

## 3. Results and Discussion

### 3.1. Temporal Variations of Ambient O_3_ and Related Parameters

#### 3.1.1. Seasonal Variation

**Figure 2:**
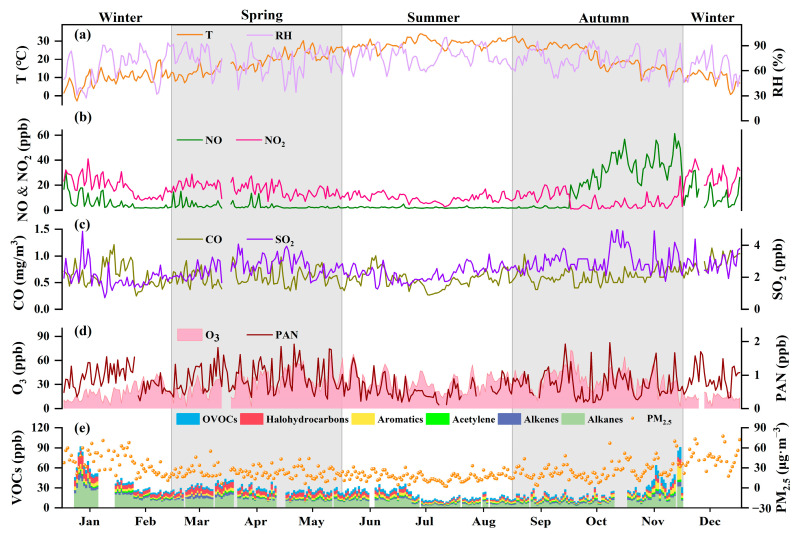
The daily mean of O_3_, PAN, VOCs, NO_x_, CO, SO_2_, PM_2.5_, and meteorological parameters (T and RH) from January–December 2021. (**a**) Time series of T and RH, (**b**) Time series of NO and NO_2_, (**c**) Time series of O_3_ and PAN, (**d**) Time series of O_3_ and PAN, (**e**) Time series of VOCs and PM_2.5_. The whole year is divided into four seasons.

The temporal variations and statistical description of each observed element during the observation period are displayed in Figure 2 and Table 1, respectively. The measured daily mean concentrations of O_3_ ranged from 2.40 to 72.76 ppb, with an annual average of 30.27 ppb, which was higher than those reported in other cities such as Xiamen (28.11 ppb) [27], Shenzhen (27.3 ppb) [28], and Melbourne (20 ppb) [29]. The annual levels of PAN, VOCs, NO, NO_2_, and SO_2_ were 0.81 ppb, 26.18 ppb, 9.18 ppb, 13.82 ppb, and 2.55 ppb, respectively. The levels of PM_2.5_ and CO were 26.66 μg·m^−3^ and 0.62 mg·m^−3^, respectively.

The mean concentration of O_3_ in autumn (36.16 ppb) was significantly higher than in all other seasons, with the maximum daily concentration also occurring in autumn (72.76 ppb), unlike in Chengdu and Beijing, but the same as the previous result from Shanghai in the YRD [17,27,30]. It reflects the local synoptic flow pattern, which is the product of the interaction of the East Asian monsoon, tropical cyclones, and the land–sea breezes over the YRD [31]. The average O_3_ concentration was the lowest in winter, which was due to weaker photochemical reactions at low ultraviolet radiation. Figure 2 shows there is a significant correlation between O_3_ and PAN (*p* < 0.05), but the lowest mean PAN concentration occurred in summer (0.59 ppb). It was explained by the location of the observatory in the YRD, which was influenced by the East Asian summer monsoon that brought clean, humid air masses and diluted PAN, none of which were conducive to photochemical production (Li and Fan, 2022). It is noteworthy that the PAN had the highest average level in the spring (0.94 ppb), which was inconsistent with some previous reports [32,33]. We attribute this to low photodegradation efficiency and accumulation of long-term non-methane volatile organic compounds (NMVOCs) in the free troposphere during winter [34,35]. Photochemistry became active in early spring and accumulated NMVOCs promoted PAN accumulation, resulting in the highest PAN levels in spring, matching the mean level of VOCs in this study, which was highest in winter (39.03 ppb).

The averaged values for PM_2.5_, NO_2_, and CO were significantly higher in winter than in other seasons, at 40.87 ppb, 21.46 ppb, and 0.71 ppb, respectively. It could be caused by weak convection in the winter, leading to higher concentrations of accumulated pollutants [36]. NO and SO_2_ were concordant with O_3_, with average concentrations highest in the autumn. Meanwhile, the ratio value of NO/NO_2_ was greater than 1.0 (3.05), indicating that less O_3_ consumption occurred in the NO_2_ photolysis cycle in autumn [37].

#### 3.1.2. Diurnal Variation

**Figure 3:**
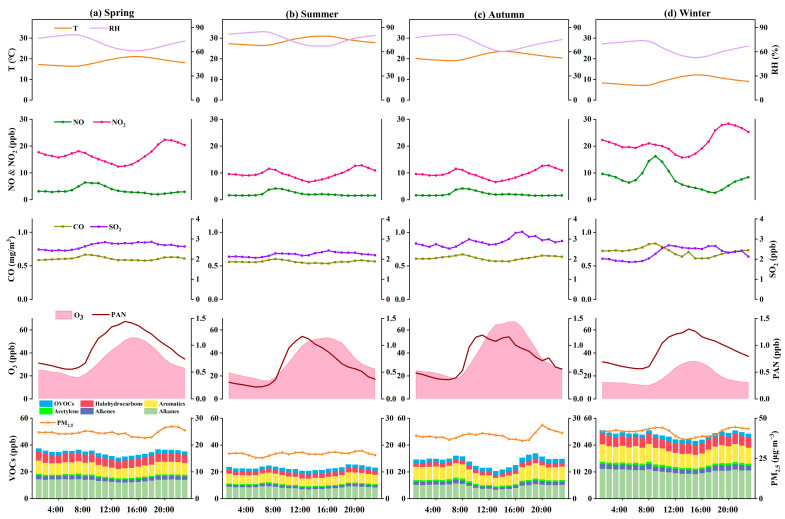
Diurnal trends of O_3_, PAN, VOCs, NO_x_, CO, SO_2_, PM_2.5_, and meteorological parameters (T and RH) in (**a**) spring, (**b**) summer, (**c**) autumn, and (**d**) winter, respectively.

The average diurnal variation patterns for O_3_, PAN, and some other pollutants as well as meteorological parameters during the 4 seasons of 2021 are shown in Figure 3. O_3_ as a secondary pollutant showed the highest value during the early afternoon (15:00–16:00) and the lowest value at 07:00–08:00. Temporal variations in solar radiation and temperature were considered major drivers of such diurnal variations in O_3_ levels [2]. PAN has a similar diurnal pattern to O_3_, reaching a maximum between 11:00 and 14:00 in all seasons, then decreasing during low solar radiation, and a minimum in the early morning (06:00–08:00, indicating the dominance of local photochemistry during the observation period [11]. Specifically, the PAN peak usually occurred 1 h earlier than that of O_3_, presumably resulting from the increasing decomposition rate of PAN with increasing temperature [27]. The variation between maximum and minimum values of PAN in summer was the highest (0.94 ppb) while was the smallest difference in winter (0.70 ppb), which was a net growth pattern that also indicates that the lifetime of PAN increases with decreasing temperature.

Contrastingly, NO_x_, CO, and VOCs levels showed a diurnal pattern opposite to O_3_ (Figure 3). The diurnal variation of NO_2_ exhibited a bimodal distribution, with a peak in the morning, followed by a decrease in NO_2_ concentration due to photolysis, and subsequent accumulation of NO_2_ at night due to primary emissions. The peak NO_x_ and CO levels in the morning were strongly related to vehicle emissions during the morning rush hour. The trend of VOCs concentration was the same as the daily variation of NO_2_, with a higher concentration in the morning, followed by a gradual decrease, but then a higher concentration at night, which was associated with the lower photochemical losses at night and the accumulation of primary emissions of pollutants. Anyway, a close correlation between precursor emissions and human activities (e.g., transportation) can be seen in the observed areas.

### 3.2. The Influencing Factors of O_3_ Using the GAM

**Figure 4:**
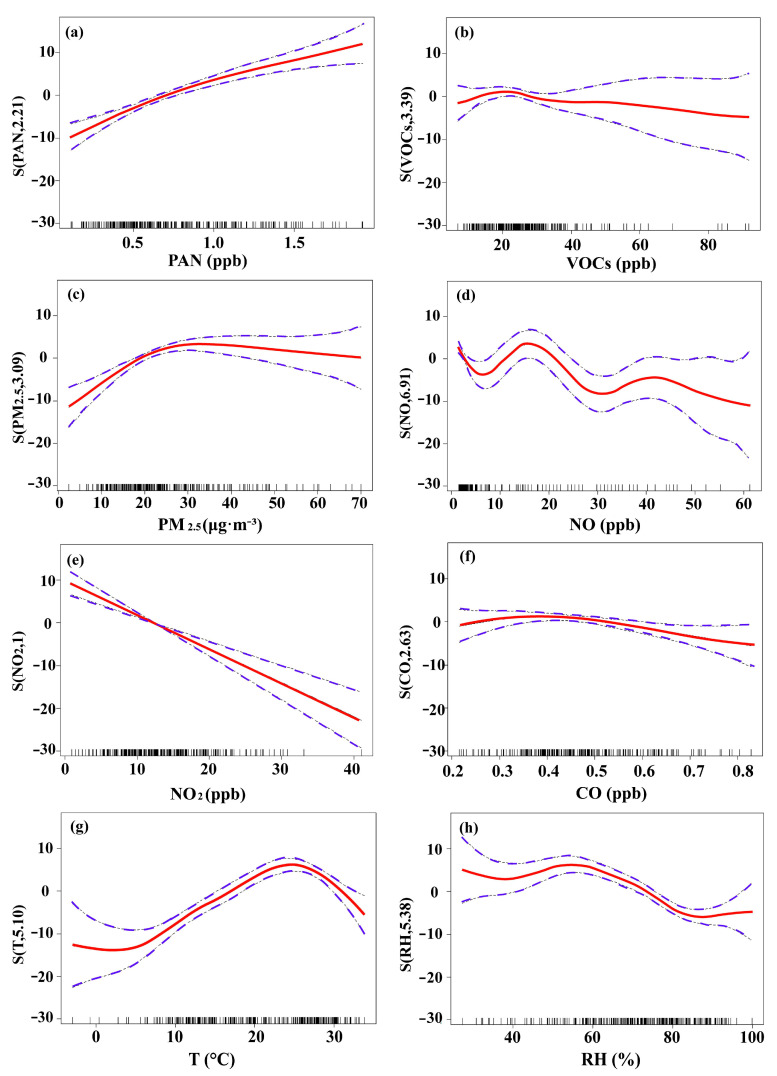
Response curves in the multiple-factor model of O_3_ to changes in (**a**) PAN, (**b**) VOCs, (**c**) PM_2.5_, (**d**) NO, (**e**) NO_2_, (**f**) CO, (**g**) T, and (**h**) RH. The y axis shows the smoothing function values. For example, S (PAN, 2.21) shows the trend in PAN when O_3_ changes, and 2.21 is the degree of freedom. The *x* axis is the influencing factor. Note that each marginal effect is denoted by a solid red line with a 95% confidence interval (purple dashed lines), and the vertical lines adjacent to the lower x-axis represent the distributions of these covariates.

Eight parameters were selected as explanatory variables (PAN, VOCs, PM_2.5_, NO, NO_2_, CO, T, RH) and O_3_ concentration as the response variable. The multi-factorial correlation analysis was performed using the GAM and the results are shown in Figure 4 and Table 2. $g\left(O_{3}\right)=32.56+f_{1}\left(NO_{2}\right)+f_{2}\left(PAN\right)+f_{3}\left(T\right)+f_{4}\left(RH\right)+f_{5}\left(PM_{2.5}\right)+f_{6}\left(NO\right)+f_{7}\left(CO\right)+f_{8}\left(VOCs\right)+0.35$ is the parameterized formula.

In accordance with the F values, explanatory variables over the monitoring period were in the order of NO_2_ (47.88) > PAN (24.22) > T (22.34) > RH (13.22) > PM_2.5_ (9.12) > NO (4.85) > CO (3.25) > VOCs (1.05). Notably, there was a significant negative correlation between NO_2_ and O_3_ (Figure 4e), which was consistent with previous results from Beijing [16], but the degree of freedom (df) of NO_2_ in this study was 1, indicating that a large proportion of O_3_ was directly produced by NO_2_ photolysis [38]. The effect of PAN on O_3_ was also not negligible, showing a nonlinear positive correlation between the two with a narrow confidence interval (CI) (Figure 4a). In general, PAN inhibits O_3_ formation by competing with O_3_ precursors and terminating free radical chain reactions [11]. However, the positive correlation results implied that PAN may also promote O_3_ production by providing more RO_2_ radicals and increasing the oxidation capacity of the atmosphere in the presence of sufficient NO_x_ [33]. Therefore, controlling vehicle emissions can reduce NO_x_ levels and effectively mitigate the O_3_-promoting effect of PAN.

The Edf of T and RH were both greater than 1 (Figure 4g,h), demonstrating a non-linear relationship with the response variable. When T < 27 °C, the O_3_ markedly increased with rising T, implying that a certain range of heating can promote the photochemical reaction of O_3_. In contrast to the direct linear relationship of many studies [16,39], the increase in temperature above 27 °C inhibited O_3_ formation. This inhibition of O_3_ formation at high temperatures was not a coincidence, as a similar situation was observed by the University of California [40]. This phenomenon was driven by atmospheric chemistry and ecosystem-climate interactions due to the strong function of an e-folding decrease of PAN at high temperatures, as well as in areas with strong sources of isoprene and NO_x_, where chemistry is more VOCs-limited could experience a decrease in O_3_ at high levels of temperature [40,41]. In addition, high temperatures may enhance surface heat flux and convective mixing, thereby increasing the atmospheric boundary layer height and diluting the O_3_ concentration [42]. When RH < 55%, the effect of RH on O_3_ concentrations did not change significantly, and when RH > 55%, O_3_ levels decreased remarkably with the increase of RH due to the interception effect of RH on precursors and the fact that O_3_ was dissolved in atmospheric water droplets and self-degraded at high relative humidity [43].

As levels of VOCs, PM_2.5_, and CO increased, O_3_ concentrations initially increased and then gradually decreased (Figure 4b,c,f). Higher PM_2.5_ levels contributed to increased O_3_ levels through the scattering effect of PM_2.5_ in a certain range, but excessively high PM_2.5_ levels reduced terrestrial ultraviolet, leading to the inhibition of photochemical reactions and hence lower O_3_ levels [21,44]. CO and VOCs had little effect on O_3_ and it seems reasonable to assume free radical reactions with NO_x_ dominated in the region. NO displayed a complex relationship with O_3_, but was generally negatively correlated due to their susceptibility to reaction [2]. HO_2_ in a high-NO atmosphere promotes the oxidation of NO to NO_2_, and NO consumes peroxyacetyl radicals to generate NO_2_, promoting O_3_ formation [9,39]. In summary, the multifactorial GAM is more interpretable and simulates more realistic O_3_ trends in the atmosphere. It demonstrates that in the YRD NO_2_ and PAN have the greatest influence on O_3_ levels, followed by T and RH.

### 3.3. Sensitivity of O_3_ Formation

**Figure 5:**
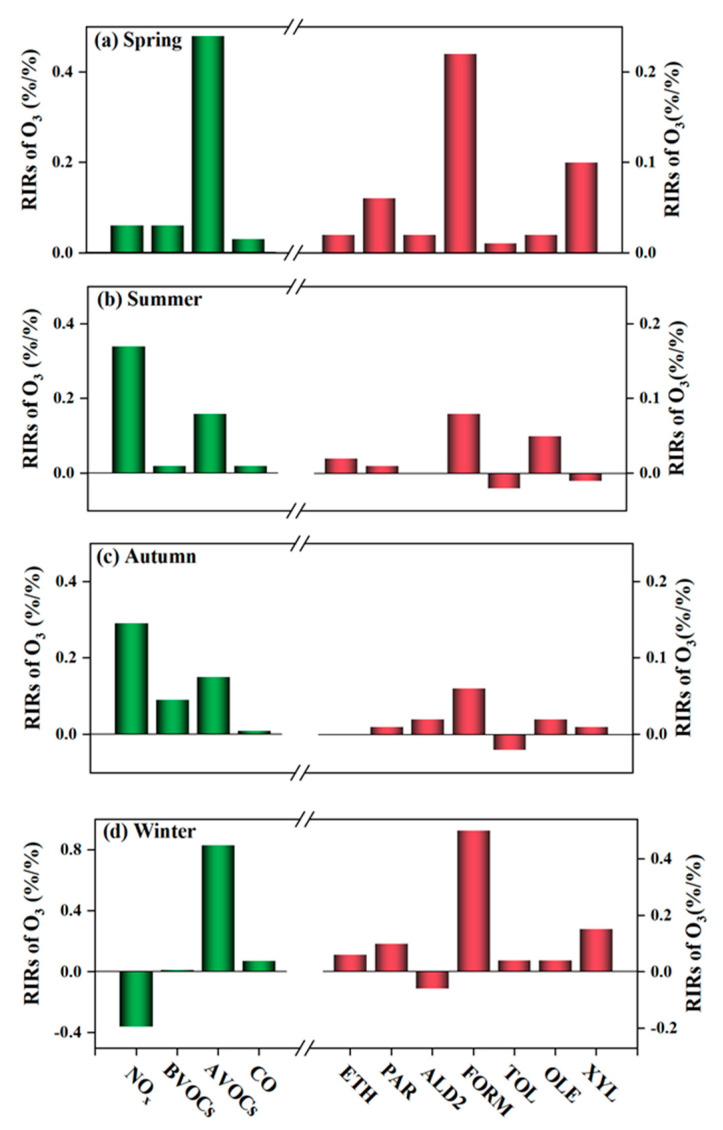
The observation-based models (OBM) calculated relative incremental reactivity (RIR) for O_3_ precursors (green) and specific species (red) in (**a**) spring, (**b**) summer, (**c**) autumn, and (**d**) winter during the daytime (07:00–19:00). BVOCs and AVOCs stand for biological VOCs and anthropogenic VOCs, respectively. ETH, PAR, ALD2, FORM, TOL, OLE, and XYL stand for ethylene, alkanes, aldehydes other than formaldehyde, formaldehyde, toluene, alkenes other than ethylene, and xylene, respectively. If the RIR value is positive, the reduction of precursors contributes to O_3_ reduction, while a negative value means that precursor reduction may lead to an increase in O_3_ concentration.

In this study, the RIR values calculated by OBM for the precursors in all seasons are shown in Figure 5. The RIR values for VOCs were significantly higher than those for NOx in spring and winter (Figure 5a,d), with a negative RIR value for NO_x_ in the winter (−0.36), indicating O_3_ production in the observing area was mainly controlled by VOCs. Notably, the RIR of BVOC (isoprene) in winter was only 0.01, which can be attributed to the fact that plant branches became bare in winter and BVOC emissions were greatly reduced, which, together with low temperatures and weak radiation in winter, caused the effect of isoprene on O_3_ formation to be lower [45]. Moreover, formaldehyde (FORM) and xylene (XYL) showed the top two RIRs for O_3_ in spring and winter, revealing their dominance in the O_3_ generation. Therefore, reducing VOCs in these two seasons is more effective for controlling O_3_ pollution. Additionally, previous studies have concluded that anthropogenic primary sources (e.g., vehicle emissions and industrial activities) contributed most to FORM in the spring and winter, that biological sources contributed more in the summer and autumn, and that the major sources of XYL were traffic and industry [46,47]. O_3_ production in summer was more sensitive to NO_x_, with a RIR of 0.34. Toluene (TOL) and XYL of AVOCs appeared to have negative values and decreases in their concentrations will instead lead to an increase in O_3_ concentrations. In autumn, O_3_ formation displayed a high sensitivity to simultaneously VOCs and NOx (RIR_(VOCs)_: 0.24, RIR_(NOx)_: 0.29), while the effect of CO on O_3_ formation was negligible (RIR_(CO)_: 0.01). Further analysis showed reducing TOL has an adverse impact on O_3_ formation and FORM needs to be prevented and controlled. In a nutshell, O_3_ formation was in the VOCs-limited in spring and winter, controlled by NO_x_ in summer, and even controlled by both VOCs and NO_x_ in autumn, and FORM emissions have to be emphasized throughout the year.

**Figure 6:**
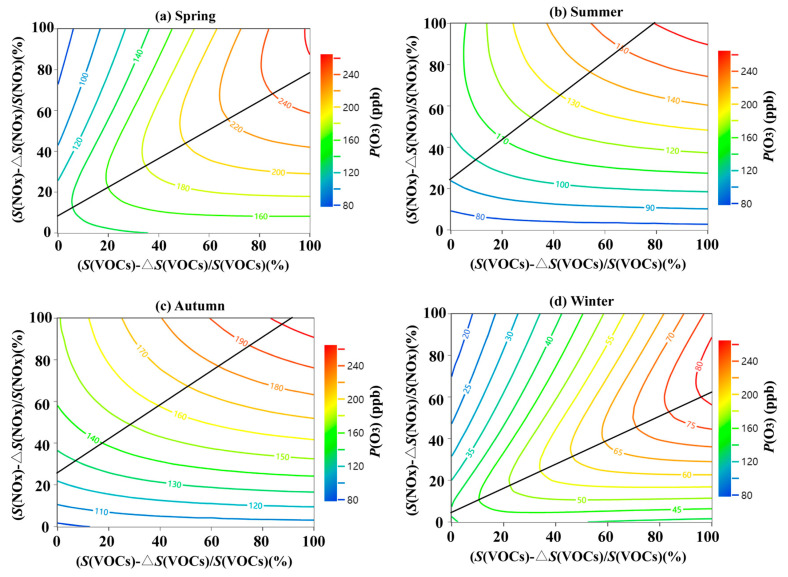
Isopleth diagrams of modeled O_3_ production potential (*P(O_3_)*) on S(VOCs) and S(NO_x_) remaining percentages (i.e., (S(VOCs)-ΔS(VOCs))/(S(VOCs)) and (S(NO_x_)-ΔS(NO_x_))/(S(NO_x_)) for four seasons in 2021 ((**a**) spring, (**b**) summer, (**c**) autumn, (**d**) winter). The black line is a ridge line.

Empirical Kinetics Modeling Approach curves were simulated and plotted to investigate the impacts of precursors reduction on O_3_ formation (Figure 6). In other words, the relationship of *P(O_3_)* with relative changes of *S*(VOCs) and *S*(NO_x_) can be expressed by isopleth diagrams for *P(O_3_)*. The mean *P(O_3_)* levels varied considerably over the four seasons, with estimates of 262 ppb, 165 ppb, 205 ppb, and 77 ppb, respectively. In spring, a 10% reduction in *S(VOCs)* resulted in a decrease of 13 ppb in *P(O_3_)*, and a 10% reduction in NO_x_ only resulted in a reduction of 2 (Figure 6a). In winter, O_3_ levels gradually decreased with an increasing reduction ratio when only VOCs was reduced; however, O_3_ levels progressively increased when only NO_x_ was reduced, especially when the reduction ratio reached to 40% (Figure 6d). It indicated that the regime was in the VOCs-limited in spring and winter as the results of the RIRs. During summer, O_3_ formation was more sensitive to NO_x_, with an increase in the percentage of NO_x_ reduction leading to a notable reduction in O_3_ levels, while VOCs reduction required a large percentage of reduction. For autumn, the *S(VOCs)* and *S(NO_x_)* data point was close to the ridge line, indicating the point was in a transition regime where significant NO_x_ reductions can be achieved in the short term but easily transition to the NO_x_-limited regime. Thus, stringent control of VOCs pollution ought to be implemented in parallel with collaborative regional prevention and control of NO_x_ to facilitate long-term control of O_3_. However, many studies have not studied the seasonal sensitivity differences in depth and finally only obtained that the study area was in the VOCs-limited control or NO_x_-limited [48,49]. Based on the above conclusions, it is necessary for YRD to dynamically adjust its prevention and control strategy in accordance with the characteristics of the O_3_ formation mechanism.

## 4. Conclusions

Long-term O_3_ observations in the YRD in 2021 displayed strong seasonal variations with a maximum in autumn (72.76 ppb) due to the metrological interaction and the lowest O_3_ level in the low-radiation winter (19.03 ppb). O_3_ levels displayed an obvious cyclic pattern of diurnal variation, with O_3_ showing the highest values in the early afternoon (15:00–16:00) due to vivid photochemical reactions and the lowest values in the 07:00–08:00. PAN presented a similar diurnal pattern to O_3_; however, the rate of decomposition of PAN increased with increasing temperature, resulting in the peak of PAN usually occurring 1 h earlier than the peak of O_3_ precursors (NO_x_, CO, and VOCs), which, in contrast to O_3_, showed a diurnal pattern, with the lowest levels in the afternoon and the maximum in the night or the morning peak.

Furthermore, GAM revealed key factors affecting O_3_ levels were NO_2_, PAN, and T. A large fraction of O_3_ was produced directly by NO_2_ photolysis, and PAN contributes to O_3_ production by providing more RO_2_ radicals and increasing the oxidation capacity of the atmosphere in the presence of sufficient NO_x_. Thus, reducing vehicle NO_x_ emissions can effectively mitigate the O_3_-promoting effect of PAN. It was found a certain temperature rise promoted the photochemical reaction of O_3_, whereas rising temperatures above 27 °C inhibited O_3_ formation. It is strongly recommended to target control in different seasons according to various O_3_ formation mechanisms. Based on the RIRs, FORM needs to be emphasized all year round.

This study extends the understanding of O_3_ pollution in the YRD region, integrates the coordinated effects of multiple parameters on O_3_ production, and quantifies the contribution of PAN to O_3_ formation for the first time, and proposes seasonal control of various precursors which are significant guidelines for photochemical pollution control in the YRD region, China.

## Figures and Tables

**Table 1:** Summary of mean concentrations of air pollutants and meteorological parameters during a full-year period in 2021.

	Mean				
	Spring	Summer	Autumn	Winter	Year
O_3_ (ppb)	32.89 ± 14.00	32.70 ± 11.41	36.16 ± 13.28	19.03 ± 9.21	30.27 ± 13.78
PAN (ppb)	0.94 ± 0.42	0.59 ± 0.30	0.75 ± 0.40	0.88 ± 0.35	0.81 ± 0.42
VOCs (ppb)	28.74 ± 5.78	19.14 ± 7.34	24.04 ± 14.80	39.03 ± 18.08	26.18 ± 13.33
PM_2.5_ (μg·m^−3^)	23.86 ± 8.83	16.74 ± 6.39	25.78 ± 12.18	40.87 ± 15.94	26.66 ± 14.36
NO (ppb)	3.47 ± 2.79	2.12 ± 0.49	23.27 ± 17.85	7.71 ± 7.29	9.18 ± 12.94
NO_2_ (ppb)	16.95 ± 5.06	9.64 ± 3.21	7.62 ± 5.88	21.46 ± 8.52	13.82 ± 8.15
SO_2_ (ppb)	2.64 ± 0.58	2.23 ± 0.44	3.03 ± 0.71	2.29 ± 0.77	2.55 ± 0.71
CO (mg·m^−3^)	0.61 ± 0.15	0.56 ± 0.15	0.62 ± 0.12	0.71 ± 0.22	0.62 ± 0.18
T (°C)	18.50 ± 5.57	28.58 ± 2.57	21.14 ± 6.36	9.45 ± 4.12	19.48 ± 8.41
RH (%)	71.68 ± 14.45	76.81 ± 10.86	72.32 ± 12.65	64.39 ± 17.19	71.33 ± 14.66

**Table 2:** The results for each variable in the GAM based on monitoring data for the full year in 2021 (estimated degrees of freedom (Edf), degree of reference (Ref. df)).

Smoothed Variables	Smooth Terms			
	Edf	Ref.df	*F* Value	*p* Value
PAN (ppb)	2.16	2.75	24.22	0.00
VOCs (ppb)	3.99	4.94	1.05	0.03
PM_2.5_ (μg·m^−3^)	3.09	3.87	9.12	0.00
NO (ppb)	6.91	7.97	4.85	0.00
NO_2_ (ppb)	1.00	1.00	47.88	0.00
CO (mg·m^−3^)	2.63	3.30	3.25	0.01
T (°C)	5.10	6.20	22.34	0.00
RH (%)	5.38	6.51	13.22	0.00
Deviance explained (%) = 83 %, Adjust R^2^ = 0.80				

## Data Availability

The data presented in this study are available on request from the corresponding author. The data are not publicly available due to privacy.
